# Current updates and future perspectives in the evaluation of azoospermia: A systematic review

**DOI:** 10.1080/2090598X.2021.1954415

**Published:** 2021-07-22

**Authors:** Nahid Punjani, Caroline Kang, Dolores J. Lamb, Peter N. Schlegel

**Affiliations:** a Department of Urology, Weill Cornell Medical College, New York, NY, USA; b Englander Institute for Precision Medicine, Weill Cornell Medical College, New York, NY, USA; c Center for Reproductive Genomics, Weill Cornell Medical College, New York, NY, USA

**Keywords:** Male infertility, azoospermia, evaluation, genetics

## Abstract

**Objectives**: To provide a summary of the current evaluation of azoospermia and insights into future perspectives in the evaluation and counselling of men with azoospermia. **Methods**: A search of PubMed, Cochrane Reviews and Web of Science databases was performed for full-text English-language articles published between 1943 and 2020 focussing on ‘future perspectives’, ‘azoospermia’ and ‘evaluation’. **Results**: Azoospermia represents a severe form of male infertility characterised by sperm production so impaired that there are no sperm present in the ejaculate. The current evaluation of azoospermia focusses on patient history and physical examination with selected adjunctive laboratory investigations including serum hormones, a karyotype and screening for Y chromosome microdeletions. Future diagnostics are focussed on identifying the underlying genetic aetiologies for azoospermia, as well as a greater emphasis on screening for systemic illness that men with severe infertility may be predisposed to develop. **Conclusion**: Azoospermia represents an extreme form of male infertility, and evaluation relies heavily on history and physical examination, as genetic evaluations for these individuals remain limited. Future evaluation will focus on next-generation sequencing and more rigorous evaluation for possible co-existing and future risk of systemic disease.

**ABBREVIATIONS**: ADGRG2, adhesion G protein-coupled receptor G2; ASRM: American Society of Reproductive Medicine; AZF: azoospermia factor; CBAVD: congenital bilateral absence of the vas deferens; CFTR: cystic fibrosis transmembrane conductance regulator; CRKL: CRK-like proto-oncogene; E2F1: E2F transcription factor 1; HAUS7: HAUS augmin-like complex subunit 7; HR: hazard ratio; KS: Klinefelter syndrome; MAZ, MYC-associated zinc finger protein; NGS: next-generation sequencing; NOA: non-obstructive azoospermia; OA: obstructive azoospermia; RHOX: reproductive homeobox on the X chromosome; SH2: SRC homology 2; TAF7L: TATA-box binding protein associated factor 7-like; TEX11: testis-expressed 11; WES: whole-exome sequencing

## Introduction

Infertility occurs in up to 15% of couples, in which a male factor is contributory in up to 50% of cases [1, 2]. Unfortunately, the precise aetiology is commonly unknown. Male factor infertility in its most severe form is known as azoospermia, or absence of sperm in the ejaculate, and may be classified as obstructive azoospermia (OA) or non-obstructive azoospermia (NOA). The evaluation and management of patients with azoospermia has progressed significantly as we continue to understand more about the infertile male. There is growing evidence that infertile men may harbour systemic disease and therefore an in-depth evaluation of these patients is critical [3]. For example, men with a history of azoospermia are, in general, at increased risk of developing cancer, and their life expectancy is limited relative to population-based control men [4–6]. In the present review, we discuss the standard evaluation of men with azoospermia and future perspectives in the evaluation and counselling of this patient population.

## Methods

A search of PubMed, Cochrane Review, and Web of Science databases was conducted using the search terms: ‘evaluation’, ‘future perspectives’ and ‘azoospermia’. Citations were searched between 1943 and 2020. Articles that were not available as full text and not written in English were excluded.

## Results

**Figure 1.**
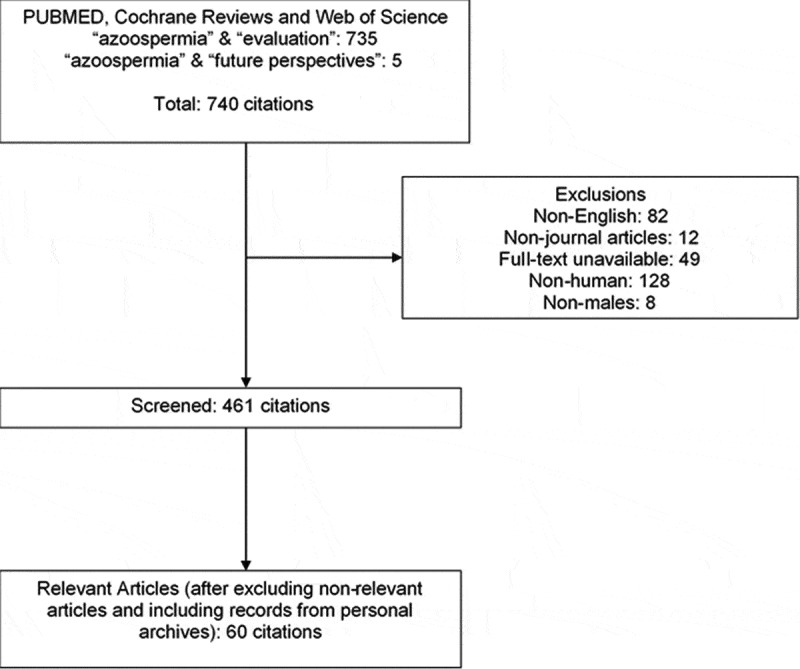
Flow chart of article selection

Our initial search yielded a total of 740 citations. In combination with a personal archive of articles, and removal of non-relevant articles, a total of 60 citations were included (Figure 1). Furthermore, the content of the relevant articles was combined with the personal experience and perspectives of the authors.

## Azoospermia

Of men with azoospermia ~40% have OA [7,8]. Generally, OA is due to the presence of an obstruction that impairs the patency of the genital tract at one or more points in the male reproductive tract. Common examples include congenital bilateral absence of the vas deferens (CBAVD), which is associated with cystic fibrosis transmembrane conductance regulator (*CFTR*) gene mutations, ejaculatory duct obstruction and/or cysts, seminal vesical atresia, idiopathic epididymal obstruction, genitourinary infections resulting in obstruction, and iatrogenic obstruction from vasectomy or other pelvic/inguinal surgery [9,10]. Most men with NOA do not have an identifiable underlying cause of impaired sperm production; in fact, for up to 60% of men with NOA, the aetiology underlying their spermatogenic failure remains unknown. Known aetiologies of NOA include numerical (e.g. Klinefelter syndrome [KS]) or structural (e.g. Y-chromosome microdeletions, azoospermia factor a [AZFa], AZFb, and AZFc; translocations and/or inversions) chromosomal anomalies, cryptorchidism, prior chemotherapy or radiation (e.g. for malignancy), hypogonadotrophic hypogonadism (e.g. Kallmann syndrome), exogenous androgen usage, iatrogenic manipulation of the pituitary gland, or secondary to infection or other insults to the testis [9,11].

## Current evaluation and diagnosis of azoospermia

The evaluation of azoospermia relies heavily on a thorough history and comprehensive physical examination to assess for possible underlying aetiologies of infertility.

### History

A focussed history remains a critical tool in the evaluation of infertile patients. Important categories include past medical history, past surgical history, developmental history, and family history of genetic conditions or infertility. A focussed infertility history should review and differentiate primary vs secondary infertility, the duration of infertility, partner history including age, and history of known infertility causes, e.g. undescended testis, childhood illness, pelvic or inguinal surgery, genitourinary surgery (i.e. vasectomy), trauma, infection, gonadotoxins, exogenous androgen replacement, and known genetic conditions. A sexual history should accompany the infertility history and should investigate erectile function, ejaculatory function, libido, frequency of intercourse, use of lubricants, and contraception. The history should further explore any areas of systemic disease or conditions.

### Physical examination

The physical examination should include a general survey to evaluate for gross abnormalities, syndromic phenotypes, under-masculinisation and general appearance, which may provide insight into other possible systemic conditions. An abdominal examination and focussed genitourinary examination should be completed. The testicles should be examined for size, consistency, and presence of masses. The spermatic cord should be evaluated for hernia, varicocele, and palpable vasa, and the penis should be assessed for meatal abnormalities, circumcision, and development. The epididymis should be evaluated for fullness or cysts, and a DRE should be performed if indicated [7,12].

Most men with OA will have a testis length of >4.6 cm, whereas those with NOA will generally present with softer testes measuring <4.6 cm [13]. OA should be suspected in men with impalpable vas deferens, a full epididymis, and/or low volume, acidic ejaculates. Men lacking palpable vas deferens require sequencing of the entire *CFTR* gene for mutations as the most common mutations causing isolated CBAVD are not normally measured in routine *CFTR* mutation screening assays. Their partners also should be tested to determine carrier status for *CFTR* mutations, and ideally the entire gene also should be sequenced to identify less common damaging mutations. Abdominal imaging may be completed to rule out any renal anomalies (more common in the case of unilateral absence of the vas deferens with no identifiable *CFTR* mutation). An X-linked form of CBAVD is now known to be caused by damaging mutations in the adhesion G protein-coupled receptor G2 (*ADGRG2)* gene, which also is clinically assessed in some selected medical genetics laboratories [14].

### Semen analysis

Semen analysis is one of the first laboratory diagnostic tests performed as part of an infertility evaluation. The AUA practice guidelines recommend two separate analyses of semen for the initial evaluation of an infertile man [15]. Although abnormal semen parameters do not necessarily indicate infertility, the complete absence of sperm in the ejaculate precludes a man from conceiving a child naturally. A diagnosis of azoospermia requires confirmation of a lack of sperm in the ejaculate following an analysis of the pellet from two separate centrifuged semen samples [12]. The WHO defined the normal ranges for semen parameters derived from analysing semen of a cohort of fertile men [16–18]. Critical components of a thorough semen analysis are shown in Table 1 [17].

**Table 1.** WHO 2010 fifth edition normal range of semen analysis parameters [17]

Semen analysis parameter	WHO 2010 (5th percentile)	Abnormality
Volume, mL	1.5	Aspermia (0 mL)
Total sperm count (TSC), ×10^6^	39	Oligozoospermia (TSC <15 × 10^6^) Severe Oligozoospermia (TSC <5 × 10^6^) Azoospermia (TSC = 0)
Concentration, ×10^6^ sperm/mL	15	
Progressive motility, %	32	Asthenospermia
Total motility, %	40	
Normal morphology, %	4	Teratozoospermia
Vitality, %	58	Necrozoospermia (100% dead)
Leucocyte count, ×10^6^/mL	<1.0	Leucocytospermia (>1 × 10^6^/mL)

The presence of abnormal semen parameters can prompt further clinical evaluation in infertile men. For example, a man with ejaculatory duct obstruction may have a semen sample characterised by azoospermia, low volume (typically <1 mL), acidic pH (due to the lack of fluid from the seminal vesicles that normally has a basic pH), and lacking fructose (produced by the seminal vesicles and may be absent if significant blockage of the seminal vesicles is present). On the other hand, a man with NOA will typically present with completely normal seminal fluid in terms of volume, pH, fructose levels, but with azoospermia.

### Laboratory investigation

As per AUA and American Society of Reproductive Medicine (ASRM) Male Infertility Practice Guidelines, the initial laboratory evaluation for men with infertility includes serum testosterone and FSH levels [12]. Other hormones, such as luteinising hormone (LH), oestradiol, prolactin and/or thyroid stimulating hormone (TSH), may be ordered as clinically appropriate [19]. Most patients with NOA will have elevated FSH levels of >7.6 mIU/mL [13]. Patients with NOA or severe oligozoospermia (<5 million sperm/mL) also should undergo a karyotype and Y-chromosome microdeletion assessment to identify genetic aetiologies such as KS (the most common numerical chromosome abnormality, 47 XXY), Robertsonian translocations or an AZF microdeletion (AZFa, AZFb, AZFc or partial deletion) [12]. Identifying the specific AZF deletion is important because complete AZFa and AZFb microdeletions have no chance of surgical sperm retrieval, whereas men with AZFc microdeletions often have sperm in their ejaculate and have sperm retrieval rates of up to 70% [20,21].

### Radiological investigation

Limited methods and indications exist for imaging in the azoospermic male. The AUA/ASRM Guidelines do not recommend either scrotal ultrasonography or TRUS in the initial infertility evaluation but do recommend renal ultrasonography in males with vasal agenesis [22].

## Future perspectives in azoospermia

### Diagnostic and evaluation tools

The diagnostic evaluation of men with azoospermia is challenging. A strong emphasis must be placed on a thorough and comprehensive medical history as it identifies many conditions that are associated with NOA. Therefore, the evaluation of azoospermic men mainly focusses on further characterising the aetiology of azoospermia to obtain additional prognostic information that may be used at the time of counselling.

### Karyotyping

In addition to the use of karyotyping to look for abnormalities such as KS, chromosomal translocations may provide further insight into the aetiology of azoospermia. In general, infertile men have a nine-times greater rate of chromosomal translocation (balanced, unbalanced and possible mosaic) compared to the general population [63]. Robertsonian translocations are the most common type of translocation consisting of a specific subgroup of autosomal structural abnormalities that occur between the five acrocentric chromosomes (13, 14, 15, 21, and 22) that are typically associated with oligozoospermia and NOA (although some men have normal semen parameters), which result in unbalanced translocations, mono- or trisomy, uniparental disomy in the offspring, and spontaneous miscarriage [65].

### Next-generation sequencing (NGS)

NGS using massively parallel sequencing technologies to obtain large amounts of individual genetic information allows subsequent application of bioinformatic approaches to define previously unrecognised genes that when defective may be associated with male infertility [23]. Using whole-exome sequencing (WES) of consanguineous families from an endogamous population, additional genes related to NOA have been detected [24]. One such example is the non-synonymous mutation of neuronal PAS 2 domain (*NPAS2)*, which interacts with circadian locomotor output cycle kaput (*CLOCK)* and was identified by WES in a consanguineous family from Turkey with NOA [25]. A total of 28 different genes involved in male infertility have been detected by NGS, of which mutations of 18 result in decreased sperm count (i.e. azoospermia or oligozoospermia; Table 2) [23]. Ongoing international collaborative studies defined more than two dozen additional novel genes purported to be associated with NOA or severe oligozoospermia and Genecards lists nearly 1000 genes associated with NOA identified using a variety of technical approaches [26,27]. At some time in the future, point of care methods may be developed to identify and categorise the genetic aetiologies of men with idiopathic NOA [24]. This remains an exciting area of research investigation.

**Table 2.** Genes defects identified in men with NOA using NGS technologies [23]

Familial	Sporadic
*DNAH6*	ADGRG2*
*MAGEB4*	CFTR*
*MEIOB*	*DNMT3L*
*NPAS2*	*HLA-DQA1, HLA-DRB1*
*SPINK2*	*SIRPA*
*SYCE1*	*SIPRG*
*TAF4B*	*SYCP3*
*TDRD9*	
*TEX14*	
*TEX15*	
*ZMYND15*	

*Causes OA.

### Future genetic tests for NOA aetiologies

One example of a specific gene associated with azoospermia due to meiotic arrest is the testis-expressed 11 (*TEX11)* gene, located in the Xq13.2 locus that may soon be routinely used for diagnosis of men with NOA. A normal TEX11 protein is essential for meiotic recombination and thus when damaging gene mutations or gene dosage abnormalities caused by microdeletions are present, meiotic arrest and azoospermia may result [28,29]. Another three examples of genes required for normal spermatogenesis include: the reproductive homeobox on the X chromosome (*RHOX*) gene, which is expressed in Sertoli cells that when defective may result in severe oligozoospermia; the TATA-box binding protein associated factor 7-like (*TAF7L)* gene, which encodes a transcription factor that when defective is associated with spermatogenic failure; and the HAUS augmin-like complex subunit 7 (*HAUS7*) gene, which encodes a protein that plays roles in centrosome regulation and cytokinesis and is associated with severe oligozoospermia [30,31].

### Experimental techniques for restoring fertility in NOA

Men with NOA must undergo surgical sperm retrieval and intracytoplasmic sperm injection in order to father biological children, and the live birth rate is ~25% [32]. Artificial intelligence and predictive modelling may aid in increasing the sperm retrieval rate in men with NOA [33]. Additionally, several promising experimental techniques to restore fertility in men with NOA are currently in the research ‘pipeline’, including transplantation of spermatogonial stem cells, differentiation of adult or embryonic stem cells, and gene therapy [34]. Currently, these techniques have only been tested in animal models and work in humans has yet to be performed [34].

## Infertility as a surrogate for systemic disease

There is evidence that infertile men have poorer overall health, increased risk of malignancy, numerous systemic conditions and autoimmune disorders. These men may also harbour genetic conditions where male infertility may be the initial presenting phenotype. Thus, growing evidence suggests that a thorough evaluation of these men at the time of presentation is key to diagnosing any systemic diseases that may worsen or progress over the course of their lifetime.

### General health

Men with infertility have overall poorer health and increased morbidity as defined by higher Charlson Comorbidity Indices, regardless of the aetiology of their infertility [5,35–37]. Furthermore, registry studies show that the cumulative quantity of semen parameter abnormalities are associated with increased risk of death [38]. The underlying reasons for poorer health in these men are still not fully understood; however, some authors suggest that altered hormone profile and hypogonadal states, different lifestyle choices, and other unknown systemic illness, as possible contributing factors [6,39]. For example, infertility has been associated with high-risk behaviours such as drug abuse (hazard ratio [HR] 1.67) and alcohol abuse (HR 1.48) [40]. However, in at least one study, the decrease in mortality among men with good semen quality was due to a decrease in a wide range of diseases and was found among men both with and without children; therefore, the decrease in mortality could not be attributed solely to lifestyle and/or social factors [6].

### Malignancy

A relationship between testicular cancer and infertility is well-established, where biopsies from infertile men demonstrated carcinoma *in situ* and subsequently a subset of these patients developed invasive germ cell malignancy [41]. Although variable, some authors report up to 20-times increased rates of testis cancer in infertile men [42]. Prostate cancer is another urological malignancy that may be associated with infertility, although the data linking the two conditions remains controversial, ranging from 50% risk reduction to 80% risk increase for infertile men developing prostate cancer [43–46]. However, high-grade prostate cancer (Gleason score 8–10) is more clearly associated with infertility [47]. Some investigations suggest there is an increased risk of bladder cancer (2.29 times) in infertile men [34].

Infertility is also associated with other non-urological malignancies. Men with azoospermia as compared to non-azoospermic infertile men had a 2.2-times greater risk of developing any malignancy [4]. One study reported increased risks of multiple malignancies in infertile men including haematological malignancy (leukaemia: 1.82-fold; non-Hodgkin lymphoma: 1.76-fold; Hodgkin lymphoma: 1.67-fold; thyroid cancer: 1.52-fold; and melanoma: 1.37-fold) [44].

### Systemic disease

Infertility is also associated with a higher risk of several systemic diseases such as cardiovascular disease (ischaemic heart disease, HR 1.48) and diabetes (HR 1.30), which may be secondary to hypogonadism and hormone disequilibrium [40]. Infertile men are also at increased risk of developing various autoimmune conditions such as rheumatoid arthritis (HR 1.91), multiple sclerosis (HR 1.91), psoriasis (HR 1.28), Grave’s disease (HR 1.46), thyroiditis (HR 1.60), and other general immune disorders, such as systemic lupus erythematous (HR 3.11) [48].

### Genitourinary birth defects

Cryptorchidism, the most common congenital genitourinary anomaly, is a well-known cause of male infertility and spermatogenic failure. However, recent research shows that a range of different types of genitourinary birth defects may also be associated with male infertility and thus, these birth defects are a component of syndromic conditions. Three genes associated with genitourinary birth defects and male infertility are MYC-associated zinc finger protein (*MAZ)*, CRK-like proto-oncogene (*CRKL*), and E2F transcription factor 1 (*E2F1*), which are discussed in this section. The discussion is not inclusive as there are other genes identified that may cause genitourinary birth defects and there are several candidate genes that require further investigation (Table 3) [49,50].

**Table 3.** Various known genes and potential gene hotspots for genitourinary birth defects [49,50]

Gene	Location	Possible genitourinary defects
cAMP-response element binding protein *(CREBBP)*	16p13	Bilateral cryptorchidism
Dual specificity tyrosine-phosphorylation-regulated kinase 1A *(DYRKA1)*	21q3	Micropenis, chordee, hypospadias, congenital anomalies of the kidney and urinary tract
EYA transcriptional co-activator and phosphatase 1 *(EYA1)*	8q13	Hypospadias, renal anomalies
Fibroblast growth factor receptor 2 *(FGFR2)*	10q26	Urethral formation, hypospadias, cryptorchidism, reduced testicular size, and renal anomalies
Hepatocyte nuclear factor 1β *(HNF1B)*	17q12	Renal anomalies, cryptorchidism, vasal agenesis, epididymal cysts, hypospadias, and asthenospermia
Insulin-like peptide 3 *(INSL3)*	19p13	Cryptorchidism
Kidney ankyrin repeat-containing protein 1 *(KANK1)*	9p23	Cryptorchidism, micropenis, hypospadias, urethral and scrotal development, and renal anomalies
Potassium channel tetramerization domain containing 13 *(KCTD13)*	16p11.2	Cryptorchidism, hypospadias, micropenis, and VUR
Methyl CpG binding protein 2 *(MECP2)*	Xq28	Cryptorchidism, hypospadias, hydronephrosis, urethral abnormalities
Orthodenticle homeobox 1 *(OTX1)*	2p15	Micropenis, abnormal scrotum, cryptorchidism, small testis, bladder exstrophy, epispadias, and renal anomalies
Paired box gene 2 *(PAX2)*	10q24	Renal anomalies, vesicoureteral reflux, cryptorchidism
Phosphoinositide-3-kinase regulatory subunit 1 *(PIK3R1)*	5q13	Cryptorchidism and decreased testicular size
RNA binding FOX-1 homolog 2 *(RBFOX2)*	22q12	Renal development, hypospadias, scrotal development
SH2B adaptor protein *(SH2B1)*	16p11.2	Hypospadias
Vesicle-associated membrane protein *(VAMP7)*	Xq28	Hypospadias, reduced penile length and cryptorchidism

### MAZ

*MAZ*, once thought to be an insignificant ‘housekeeping’ gene, is located at 16p11.2 of the human genome and encodes a C2-H2 zinc finger transcription factor involved in the Wnt signalling pathway [51]. *MAZ* is expressed throughout the body and associated abnormalities occur when either chromosomal microdeletions or microduplications alter this dosage-sensitive gene and the resultant amount of protein made in the cell (meaning too little or too much protein expression causes a pathology) [51]. Genitourinary defects present in individuals diagnosed as non-syndromic (i.e. with one isolated anomaly), occurred in haploinsufficient males resulting in cryptorchidism, micropenis, kidney, and bladder abnormalities [51]. Individuals with abnormal copy number variants of *MAZ* (duplications or too much protein vs deletions or too little protein) also have other systemic abnormalities including: behavioural and cognitive abnormalities, gastrointestinal, dermatological, ocular, and cardiac manifestations [3,52,53].

### CRKL

*CRKL* encodes the SRC homology 2 (SH2) and SH3 homology adaptor protein that plays a role in mediating tyrosine kinase signalling pathways [54]. This well-known gene causes many of the major anomalies present in 22q11.2 deletion syndrome (DiGeorge syndrome) and was more recently defined as the gene-dosage defect that causes the genitourinary abnormalities found in both DiGeorge syndrome and seemingly non-syndromic patients with upper and lower tract genitourinary anomalies. Associated birth defects included micropenis and cryptorchidism, but the histopathology identified in the cryptorchid mouse model was atypical spermatogenic failure unlike the spermatogenic arrest present in individuals (or mice) with cryptorchid testis [54]. This protein therefore has a unique role in testicular descent, spermatogenesis, and like *MAZ* described above is associated with numerous other systemic conditions such as cardiac, developmental, gastrointestinal, ocular, auditory, and craniofacial abnormalities [3].

### E2F1

*E2F1* encodes a transition factor that is involved in cell cycle regulation and apoptosis and located in locus 20q11.22 [55,56]. From an infertility perspective, men (and mouse models) with E2F1 overexpression from microduplications have less severe phenotypes such as hypospermatogenesis, whereas those with gene deletions have more severe phenotypes such as Sertoli-cell only, as is frequently observed in gene-dosage abnormalities [56,57]. Furthermore, copy number variants of *E2F1* are associated with cryptorchidism, which indicates that E2F1 plays a role not only in spermatogenesis, but also testicular descent [57,58]. *E2F1* gene defects (mutations or copy number variations) are also associated with other abnormalities including malignancies, renal, cardiac, stature, language and hearing, and facial abnormalities [3].

## Genetics and male infertility

Numerous genetic conditions have been associated to both infertility and azoospermia including chromosomal conditions, non-chromosomal conditions and genetic abnormalities related to disorders of sexual differentiation. There continues to be ongoing research and discovery of genes associated with infertility and spermatogenesis [59]. Table 4 [60] lists common genetic conditions associated with male infertility.

**Table 4.** Genetic conditions which negatively impact male fertility [60]

Chromosomal conditions
Y chromosome microdeletions (AZFa, AZFb, AZFc)
Structural Y-chromosome changes (isodicentric Y chromosomes)
X chromosome conditions (TEX11, MAGEB4, RHOX, HAUS7, and TAF7L)
Klinefelter syndrome (KS)
Kennedy disease
47 XYY male
46 XX male
Kallmann syndrome
Robertsonian translocations
Fanconi anaemia
β-Thalassemia
**Non-chromosomal conditions**
Primary ciliary dyskinesia
Multiple morphological abnormalities of sperm flagella
Aurora kinase C deficiency
Young syndrome
Globozoospermia
Cation channels of sperm (CATSPER)
**Disorders of sexual differentiation**
Androgen insensitivity syndrome
Gonadal dysgenesis
5α-reductase deficiency
Persistent Müllerian duct syndrome
**Other**
Myotonic dystrophy
Noonan syndrome

### The identification of genetic defects as a pathway for male infertility treatment

Many of the men identified as having single gene defects that are causally linked with severe defects in spermatogenesis present with a histological picture of maturation arrest [24]. In this condition, early germ cells are typically present in normal numbers, but development of these germ cells stops (often uniformly) at a specific developmental stage resulting in azoospermia. In many cases, expression of the defective gene occurs at or near the developmental stage where spermatogenesis is disrupted. These observations not only suggest that germ cell development was caused by the genetic variant, but also raise the potential to treat the maturation arrest condition by replacing the gene or gene product or supporting sperm development using a specific intervention based on the identified genetic defect. In this treatment approach, identification of the specific causal defect for infertility would be a critical first step in developing a personalised approach to treatment of these patients who present with vexing clinical cases that currently have a limited prognosis for sperm retrieval.

Similarly, men with an apparently uniform condition of germ cell aplasia may have had depletion of spermatogenic cells occur because of a single gene defect, or a combination of genetic factors that resulted in the ‘Sertoli cell-only’ phenotype. Great enthusiasm has been generated for a restoration of germ cells (and spermatogenesis) within these testes using stem-cell and induced pluripotent stem cell-based technologies that offer the possibility that transformation of other somatic stem cells into spermatogenic cells could be employed to re-populate the germinal epithelium and ‘fix’ the condition of germ cell aplasia. However, for the substantial number of men likely to have Sertoli cell-only because of a defect in the support, maintenance and development of germ cells, stem cell-based therapy alone would not address this condition. Specific replacement of the genetic defect or more global support of these transformed and transplanted germ cells would be required. Again, an enhanced understanding of the genetic basis of spermatogenic failure would be critical for treatment of these patients.

## Conclusion

NOA represents an extreme form of male infertility that requires a thorough history and physical evaluation. Limited current genetic evaluations are available to characterise the underlying defects in spermatogenesis for these patients. Typically, these men will require invasive surgical treatment to conceive biological children. NGS is a promising technique that can be used to identify genes associated with infertility. A thorough evaluation of infertile men with azoospermia is important to identify the presence of co-existent or future risk of systemic disease. In addition, identification of the causes of infertility may eventually lead to the development of specific therapeutic interventions for these patients with challenging defects in sperm production.
